# DDX3X loss is an adverse prognostic marker in diffuse large B-cell lymphoma and is associated with chemoresistance in aggressive non-Hodgkin lymphoma subtypes

**DOI:** 10.1186/s12943-021-01437-0

**Published:** 2021-10-16

**Authors:** Atish Kizhakeyil, Nurmahirah Binte Mohammed Zaini, Zhi Sheng Poh, Brandon Han Siang Wong, Xinpeng Loh, Aik Seng Ng, Zun Siong Low, Praseetha Prasannan, Chun Gong, Michelle Guet Khim Tan, Chandramouli Nagarajan, Dachuan Huang, Pang Wan Lu, Jing Quan Lim, Sharon Barrans, Choon Kiat Ong, Soon Thye Lim, Wee Joo Chng, George Follows, Daniel J. Hodson, Ming Qing Du, Yeow Tee Goh, Suat Hoon Tan, Nicholas Francis Grigoropoulos, Navin Kumar Verma

**Affiliations:** 1 Lee Kong Chian School of Medicine, Nanyang Technological University Singapore, 11 Mandalay Road, Clinical Sciences Building, Singapore, 308232 Singapore; 2 Department of Haematology, Singapore General Hospital, The Academia, Level 3, 20 College Road, Singapore, 169856 Singapore; 3 School of Biological Sciences, Nanyang Technological University Singapore, 60 Nanyang Dr, Singapore, 637551 Singapore; 4 Wellcome MRC Cambridge Stem Cell Institute, Cambridge, UK; 5 Clinical Translational Sciences, Singapore General Hospital, The Academia Level 9, 20 College Road, Singapore, 169856 Singapore; 6 Lymphoma Genomic Translational Research Laboratory, Division of Cellular and Molecular Research, National Cancer Centre Singapore, 11 Hospital Drive, Singapore, 169610 Singapore; 7 Haematological Malignancy Diagnostic Service (HMDS), St. James’s Institute of Oncology, Leeds, UK; 8 Cancer and Stem Cell Biology, Duke-NUS Medical School, 8 College Road, Singapore, 169857 Singapore; 9 Genome Institute of Singapore, 60 Biopolis Street Genome, Singapore, 138672 Singapore; 10 Director’s office, National Cancer Centre Singapore, 11 Hospital Drive, Singapore, 169610 Singapore; 11 Office of Education, Duke-NUS Medical School, 8 College Road, Singapore, 169857 Singapore; 12 National University Cancer Institute, Singapore, Singapore; 13 Cancer Science Institute of Singapore, National University of Singapore, Singapore, Singapore; 14 NUS Center for Cancer Research (N2CR) and Dept of Medicine, Yong Loo Lin School of Medicine, National University of Singapore, Singapore, Singapore; 15 Addenbrooke’s Hospital NHS Foundation Trust, Cambridge, UK; 16 Department of Pathology, University of Cambridge, Cambridge, UK; 17 National Skin Centre Singapore, 1 Mandalay Road, Singapore, 308205 Singapore

**Keywords:** DDX3X mutation, Hematolymphoid malignancy, Prognosis, Tumour metastasis, Drug resistance

## Main text

Non-Hodgkin a diverse group of malignancies, encompassing the common diffuse large B-cell lymphoma (DLBCL) to the rarer T-cell lymphomas. Chemoresistance is a major barrier to treatment and the mechanisms through which it occurs are incompletely understood [1]. Although efforts are made to target frequently dysregulated pathways in NHL subtypes, these diseases still evolve into aggressive forms resistant even to newer therapies [2].

DEAD box helicase 3, X-linked (DDX3X) is an ATP-dependent RNA helicase and is involved in multiple cancer-related cellular processes, including transcriptional regulation, cell adhesion, signal transduction and stemness [3]. It plays tumor suppressive or oncogenic roles depending on tumor type, and is implicated in various cancer types, including glioma, medulloblastoma, squamous cell carcinoma, hepatocellular carcinoma, lung and breast cancer [3]. A role for DDX3X in NHL subtypes remains unclear.

Here, we present our novel findings that *DDX3X* mutations are associated with poor prognosis in DLBCL. We demonstrate that mutation/loss of DDX3X in cell lines originated from DLBCL, cuteneous T-cell lymphoma (CTCL) and NK-cell/T-cell lymphoma (NKTCL) results in increased STAT3/p42/p44 phosphotylation and the development of chemoresistance. Intriguingly, DDX3X mutated/depleted B- and T-cell lineage NHL cell subtypes remain sensitive to pharmacological inhibitors of STAT3.

### Recurrent *DDX3X* mutations in DLBCL are associated with worse clinical outcomes

**Fig. 1:**
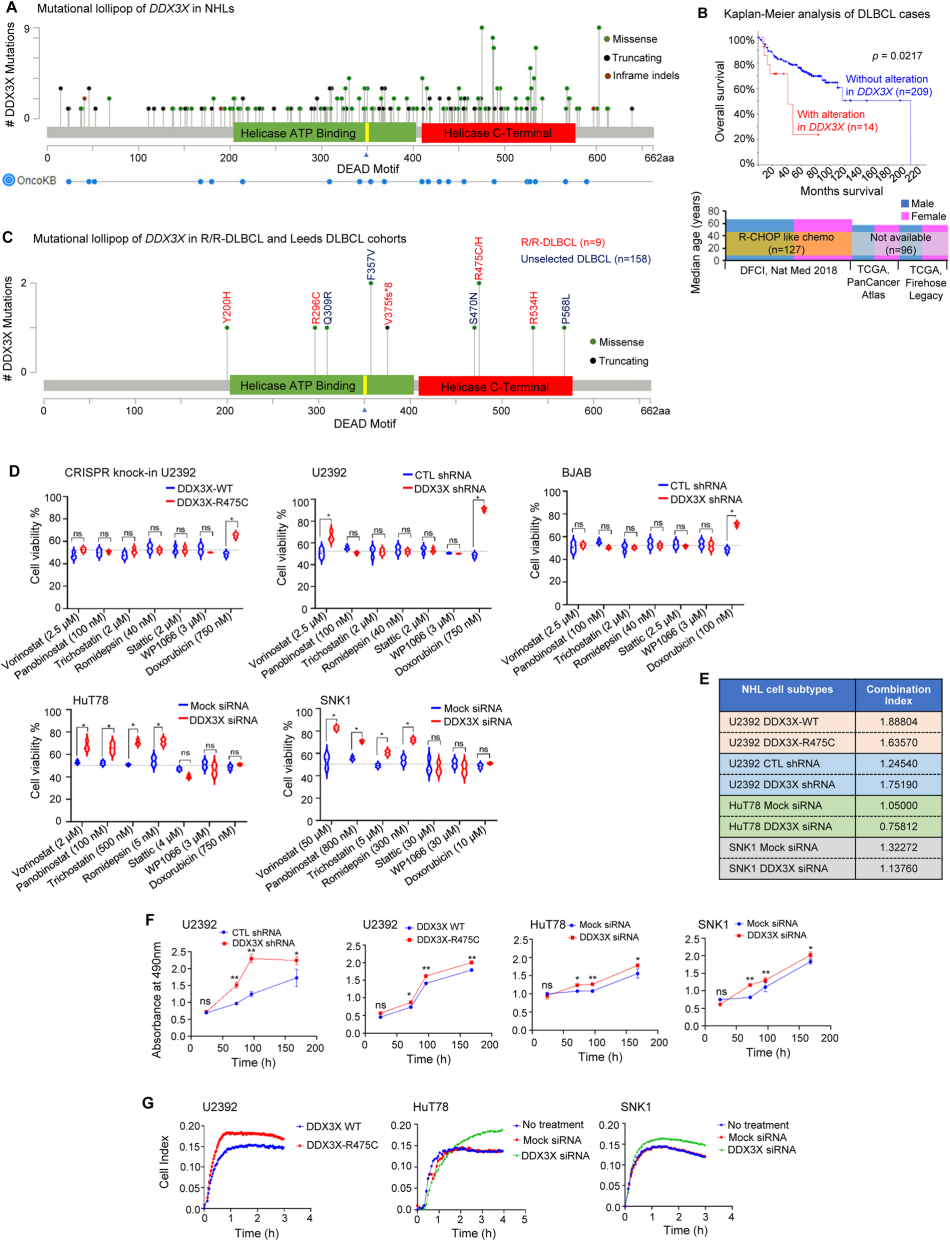
Mutational landscape of *DDX3X*, its clinical impact and the effect of DDX3X mutation/loss on proliferation, invasiveness and chemoristance in NHL cell subtypes. **A** A lollipop plot showing mutations in the *DDX3X* gene in NHL that were identified using cancer-associated genomic databases from multiple repositories (cBioPortal, ICGC, COSMIC), published literature and OncoKB. **B** Kaplan-Meier survival analysis of DLBCL patients with or without mutations in the *DDX3X* gene showing overall survival. Available data in cBioPortal are from three different cohorts - the Diffuse Large B cell Lymphoma (DFCI, Nat Med 2018; *n* = 127), Diffuse Large B-Cell Lymphoma (TCGA, PanCancer Atlas; *n* = 48), and Lymphoid Neoplasm Diffuse Large B-cell Lymphoma (TCGA, Firehose Legacy; n = 48); accessed on 14 September 2021. Patients under the the Diffuse Large B cell Lymphoma (DFCI, Nat Med 2018) cohort were treated with R-CHOP like chemotherapy, whereas treatment information for patients under the other two cohorts is not available. **C** A mutational lollipop plot depicting 6 different mutations identified in R/R-DLBCL patients (*n* = 9) using whole exome sequencing and 4 different mutations identified in unselected DLBCL patients (*n* = 158) using targeted sequencing of exons 8–15. **D** Point mutation in *DDX3X* (*DDX3X-R475C*) in U2392 cells was created using CRISPR knock-in technique and DDX3X was depleted in U2392, BJAB HuT78 and SNK1 cell lines by nucleofecting with specific shRNA or siRNA (*WT*, wild-type; *CTL,* control; *Mock,* non-specific siRNA). These cells were incubated with IC_50_ concentrations (of WT cells as indicated) of vorinostat, panobinostat, trichostatin, romidepsin, stattic, WP1066, or doxorubicin for 48 h. Cell viability was accessed by the MTS-based assay. **E** Cells, as described in “D”, were treated with a varying combinations of vorinostat and WP1066 and their effects on cell viability were determined and tabulated in terms of “Combination Index”. **F** The effect of DDX3X mutation/knockdown on cell proliferation rates in U2392, HuT78 and SNK1 cells were determined by measuring cell viability using MTS-based assay at multiple time-points up to 1 week. (**G**) Control or DDX3X-mutant/depleted U2392, HuT78 and SNK1 cells were serum starved and allowed to transmigrate through Matrigel towards 10% human serum-enriched medium in the trans-well plates for up to 4 h. Cell migration was automatically quantified using impedance-based measurements in real-time by xCELLigence Real Time Cell Analyzer. Data represent 3 independent experiments and values in graphs are mean ± SEM. **, *p* < 0.01 *, *p* < 0.05; *ns*, non-significant

Navigation through NHL patient samples in the cBioPortal [4] database revealed that *DDX3X* was mutated in 49 out of 1343 (3.6%) DLBCL cases. Moreover, available database in the ICGC [5] and the COSMIC [6] portals revealed mutations in *DDX3X* in 63 out of 1319 (4.7%) and 160 out of 5160 (3.1%) DLBCL cases, respectively. Besides DLBCL, *DDX3X* was also recurently mutated in other NHL subtypes, including Burkitt lymphoma (BL) and NKTCL [7]. Altogether, we identified 165 missense, 81 truncating, and 3 in-frame indel mutational variants in the *DDX3X* in NHL subtypes (Fig. 1A). DLBCL patients with *DDX3X* mutations (14 cases out of 223 from 3 different cohorts) had significantly worse median overall survival (41.13 months) compared to cases with wild-type *DDX3X* (211.07 months) (Fig. 1B). The 5-year overall survival of DLBCL patients with *DDX3X* lesions was only 22% compared to 72% (*p* = 0.021) for patients without *DDX3X* alterations (Fig. 1B). Data availability for other end-points, such as progression-free survival or event-free survival, was insufficient for a robust analysis. Notably, we found several other mutations that co-existed in DDX3X mutant DLBCL cases (Table S2).

We performed whole exome sequencing and detected *DDX3X* mutations in 4 out of 9 relapsed/refractory DLBCL patients treated with R-CHOP or similar regimens. Five damaging *DDX3X* mutations were identified (PolyPhen-2 score = 1, Table S1) in the catalytic domains - R296C and V375fs*8 in the “helicase ATP-binding” and R475C, R475H and R534H in the “helicase C-terminal” (Fig. 1C). Targeted sequencing of *DDX3X* on exons 8–15 in samples from 158 unselected DLBCL patients showed damaging DDX3X variants in 5 subjects (3, 95% confidence interval 1–7%) (Fig. 1C). All the identified *DDX3X* mutations were confirmed somatic and occurred in residues that are highly conserved across various species. These data indicate that *DDX3X* mutations are important determinants of response to chemotherapy in DLBCL.

### DDX3X mutation/loss in NHL cell subtypes increases resistance to antineoplastic drugs

To determine the functional role of DDX3X mutations in drug resistance, we stably expressed mutant DDX3X-R475C in U2392 cells using CRISPR knock-in technique [8] and knocked-down DDX3X in a panel of NHL cell lines [DLBCL (U2392, BJAB), BL (Raji,), NKTCL (SNK1, SNK6, NKYS), and CTCL (HuT78, MJ, MyLa)] using DDX3X-targeted shRNA or siRNA (Fig. S1). Cells were treated with IC_50_ concentrations (of wild-type cells) of commonly used histone deacetylase (HDAC) inhibitors (vorinostat, panobinostat, trichostatin, romidepsin), STAT3 inhibitors (stattic, WP1066) or doxorubicin and cell viability was analyzed by MTS-based assay. We observed significant resistance to doxorubicin in DDX3X-mutant/depleted U2932 and BJAB cells and to HDAC inhibitors in HuT78 and SNK1 cells (Fig. 1D; Fig. S2). Importantly, DDX3X-mutant/depleted NHL cell subtypes remained sensitive to pharmacological STAT3 inhibition (Fig. 1D), although no synergism of drug action was identified in these cell-types as analyzed by determining “Combination Index” using checkerboard assay and the CompuSyn software (Fig. 1E). These results strongly support the clinical data suggesting that *DDX3X* mutations cause true chemoresistance and worse overall survival, rather than simply being surrogate markers in NHL subtypes, including DLBCL, NKTCL and CTCL.

### DDX3X mutation/loss enhances proliferation and migratory potential of NHL cell subtypes

DDX3X mutation/depletion in U2932, HuT78 and SNK1 cells significantly increased their proliferation rates in comparison to controls, as determined by MTS-based assay over 7 days (Fig. 1F). In contrast, forced overexpression of wild-type DDX3X in U2932 and HuT78 cells significantly decreased proliferation (Fig. S3). Real-time monitoring of cells transmigrating through Matrigel, using impedance-based measurements, showed significantly increased migratory/invasive potentials of DDX3X-mutant/depleted NHL cell subtypes (U2932, HuT78 and SNK1) (Fig. 1G). Western immunoblot analysis of DDX3X-mutant/depleted U2932 and HuT78 cells detected increased vimentin expression in these cell-types (Fig. S4). Overexpression of vimentin has been associated with aggressive transformation in lymphoma [9].

### DDX3X mutation/loss in NHL sybtype-derived cells increase cyclin-D1 expression

**Fig. 2:**
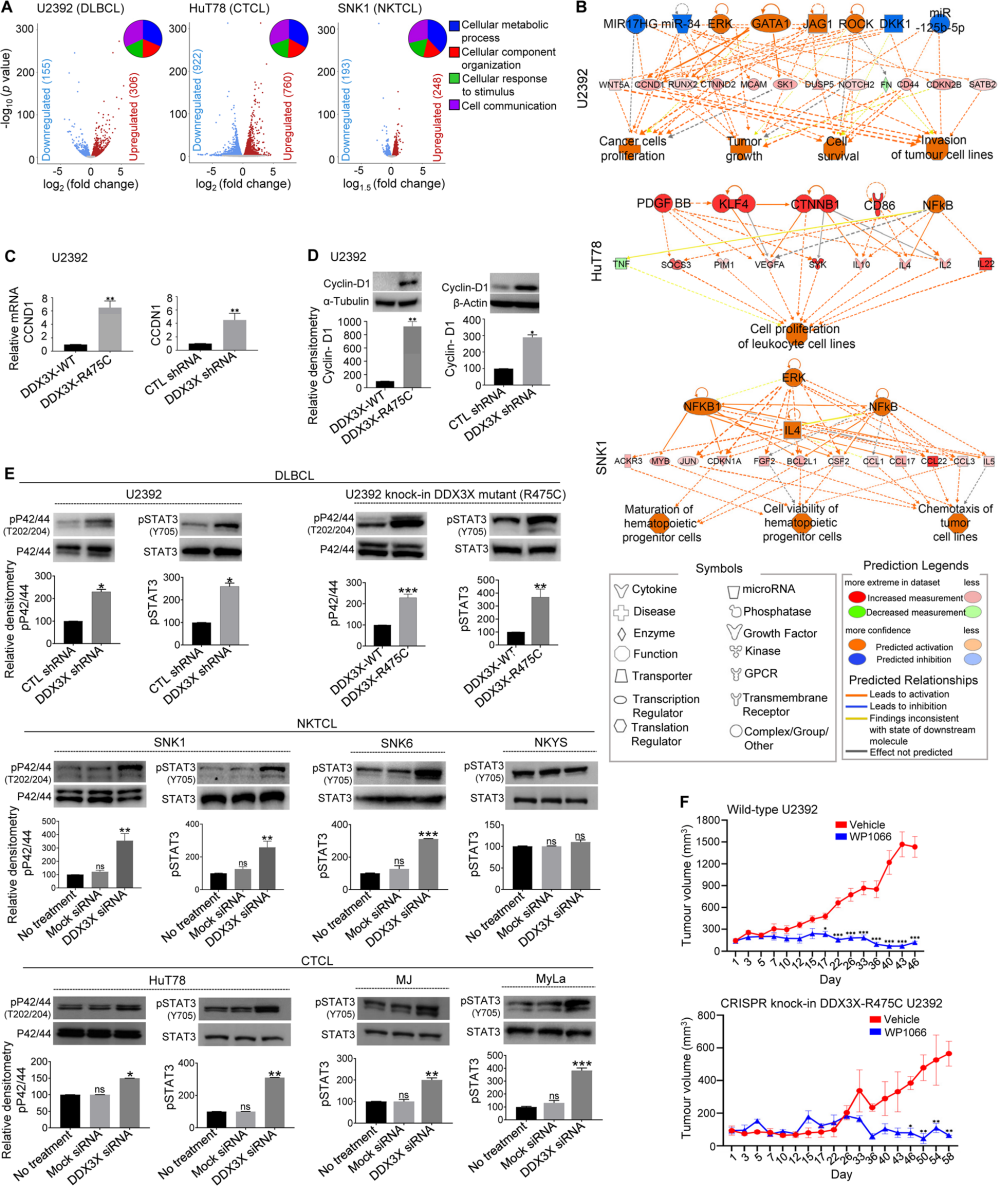
RNA-seq and protein network analysis of DDX3X mutant/depleted NHL cell subtypes and the molecular effect of DDX3X mutation/loss on DLBCL, NKTCL and CTCL cell lines. **A** Volcano plots showing DEGs in DDX3X-R475C mutant U2392, and DDX3X-depleted HuT78 and SNK1 cells (false discovery rate < 0.05 and *p* < 0.05) as analysed by RNA-seq (*n* = 3 each). Gene Ontology functional mapping of DEGs are shown in the corresponding insets. **B** Molecular interaction networks of DEGs that were identified by RNA-seq analysis of the above DDX3X-mutant/depleted cell lines, as predicted by the IPA®. Symbols representing protein types and their predicted relationships are provided in the legends. Solid and dashed lines represent direct and indirect interactions, respectively. **C** The mRNA levels of cyclin-D1 (*CCND1*) in U2392 cells expressing mutant DDX3X-R475C (*left panel*) or transfected with DDX3X shRNA (*right panel*) were quantified by RT-qPCR (*WT*, wild-type; *CTL,* control). **D** Cyclin-D1 expression levels in U2392 cells expressing mutant DDX3X-R475C (*left panel*) or transfected with DDX3X shRNA (*right panel*) were quantified by Western immunoblotting. Relative densitometry graphs quantifying cyclin-D1 expression are presented. **E** Control or DDX3X-mutant/depleted cell lines of DLBCL (U2392), NKTCL (SNK1, SNK6, NKYS) and CTCL (HuT78, MJ, MyLa) were analysed by Western immunoblotting to determine phosphorylation levels of STAT3 and p42/44. Relative densitometry graphs quantifying the expression levels of indicated proteins are presented. (**F**) U2392 cells with wild-type DDX3X or CRISPR knock-in mutant DDX3X-R475C were subcutaneously injected into NOD.Cg-Prkdc^scid^Il2rg^tm1Wjll^ mice. Cells were left to grow for 3 weeks to develop into tumors. Xenografted mice were then injected (intra-tumoral) with vehicle or WP1066 (40 mg/kg) every 3 days for up to Day-58. Tumor volume (mm^2^) on each mice was recorded twice/week by caliper measurements and calculated by the modified ellipsoidal formula. *n* = 5 under each group. Data represent at least three experiments and values in graphs are mean ± SEM. ***, *p* < 0.001; **, *p* < 0.01; *, *p* < 0.05; *ns*, non-significant

To further investigate the role of DDX3X in NHL subtypes, we carried out whole RNA-seq analysis of mutant DDX3X-R475C U2392, DDX3X-depleted HuT78 and DDX3X-depleted SNK1 cells in comparison to their wild-type and identified 451, 1682 and 441 differentially expressed genes (DEGs), respectively (Fig. 2A; Table S3-S5). We next performed gene network analysis using IPA® (a web-based application that enables analysis, integration, and interpretation of RNA-seq and other ‘omics’ datasets), DAVID (a biological module-centric algorithm that functionally analyzes large gene lists), and GSEA (an analytical method that determines whether an a priori defined gene sets shows significant differences between two biological states). This multimodal analysis of DEGs showed that DDX3X mutation/loss in NHL cells enriched cyclin-D1 and JAK-STAT3 pathways (Fig. 2B; Fig. S5; Table S6). Using RT-qPCR and Western-immunoblot analysis, we verified that cyclin-D1 mRNA and protein levels were significantly elevated in DDX3X-mutant/depleted U2392 and Raji cells relative to control (Fig. 2C,D; Fig. S6). A subset of NHL patients, including 2.1% of DLBCL cases, has been found to overexpress cyclin-D1 [10] and the upregulation of cyclin-D1 has been associated with doxorubicin resistance in gastric cancer [11].

### DDX3X mutation/loss in NHL cells enhances STAT3/MAPK activation

STAT3 and MAPK are well-studied oncogenes, known to inhibit apoptosis and decrease response to cytotoxic agents [12]. Western immunoblot analysis of DDX3X-mutated/depleted cell lines derived from DLBCL (U2932), NKTCL (SNK1, SNK2) and CTCL (HuT78, MJ, MyLa) showed significantly enhanced expression of phosphorylated STAT3 (Fig. 2E). Notably, NKYS cells express mutant STAT3 with high levels of baseline activity that did not increase further. STAT3 knockdown did not affect DDX3X expression levels nor did STAT3 directly interact with DDX3X (Fig. S7), suggesting that STAT3 hyperphosphorylation is a downstream effect of *DDX3X* lesions. Moreover, DDX3X mutation/depletion in NHL subtypes (DLBCL, NKTCL, CTCL) significantly increased the phosphorylation of p42/44 MAPK (Fig. 2E). STAT3/p42/p44 pathways are thus likely to be key mediators of the tumorigenic effects of *DDX3X* mutations in the NHL subtypes studies. Xenografted mutant DDX3X-R475C U2392 cells showed comparable sensitivity to the STAT3 inhibitor WP1066 in vivo in comparison to wild-type U2392 cells into the NOD.Cg-Prkdc^scid^Il2rg^tm1Wjll^ mice (Fig. 2F).

## Conclusions

Despite being uncommon, the biological effects *DDX3X* mutations/loss in DLBCL and other NHL subtypes (NKTCL, CTCL), suggest a worse overall survival. Given the high incidence of DLBCL [13, 14], the absolute number of patients with mutated *DDX3X* is likely to be substantial. Our results also suggest that STAT3 inhibition may be a rational choice for chemoresistant NHL (DLBCL, NKTCL, CTCL) patients with mutated *DDX3X*. The STAT3 inhibitor AZD9150 is currently in phase 1b clinical trial in a subset of patients with heavily pre-treated lymphoma [15]. Further studies are needed to improve our understanding of the consequences of *DDX3X* mutation/loss, which would help improve risk stratification of aggressive NHL subtypes and identify new therapeutic options for patients with poor prognosis.

## Supplementary Information

**Additional file 1: Supplementary Fig. 1.** RNAi-mediated depletion of DDX3X in selected NHL cell lines. The above indicated NHL cell lines and primary T-cells were nucleofected with DDX3X siRNA or Mock siRNA (control) and incubated for 72 h. Few cell lines were stably transfected with DDX3X shRNA or control (*CTL*) shRNA (as indicated) and were induced to knockdown DDX3X. In addition, U2392 cells were induced to express mutant DDX3X-R475C using CRISPR knock-in technique. (**A**) Cellular lysates from control and DDX3X-depleted/mutant cells were Western immunoblotted for quantifying DDX3X protein expression. Blots were re-probed for GAPDH or β-actin as loading control. (**B**) Relative levels of DDX3X mRNA were evaluated using quantitative reverse transcription PCR (RT-qPCR). Values in the RT-qPCR bar graphs are mean ± SEM. Results represent at least 3 independent experiments. **, *p* < 0.01. **Supplementary Fig. 2.** Consequence of DDX3X loss on refractoriness of HuT78 cells towards the effect of vorinostat. HuT78 cells were nucleofected with DDX3X siRNA or Mock siRNA (control) and after 48 h, cells were incubated with vorinostat (IC_50_). Cells were analyzed using MTS assay for cell viability (**A**) and Annexin-V/PI staining with subsequent flow-cytometry for apoptosis (**B**). Cell lysates were collected and Western immunoblotted for determining the expression levels of DDX3X, cleaved PARP, and cleaved caspase 3 (**C**). Blots were re-probed with anti-GAPDH to confirm equal loading. Values are mean ± SEM and results represent at least 3 independent experiments. **, *p* < 0.01; ***, *p* < 0.001; ****, *p* < 0.0001. **Supplementary Fig. 3.** Effect of DDX3X overexpression on the rate of proliferation of CTCL and DLBCL cells. HuT78 and U2392 cells were nucleofected with plasmid containing wild-type DDX3X (*DDX3X OE*) or vector alone. The overexpression of DDX3X in HuT78 (**A**) and U2392 (**B**) cells was confirmed by Western immunoblotting. Relative densitometry graphs of DDX3X in both cell types are shown. (**C**) The rate of HuT78 cell proliferation was determined by MTS-based assay, performed in triplicates at 48 h and 96 h. (**D**) The viability of U2392 cells was determined by MTS-based assay, performed in triplicates at 48 h. Values are mean ± SEM and results represent at least 3 independent experiments. *, *p* < 0.05; **, *p* < 0.01. **Supplementary Fig. 4.** Effect of the modulation of DDX3X expression on the expression levels of vimentin in CTCL and DLBCL cells. (**A**) HuT78 cells were nucleofected with DDX3X siRNA or Mock siRNA (control) and then lysed after 72 h. (**B**) DDX3X wild-type (*WT*, control) and CRISPR knock-in mutant DDX3X-R475C U2392 cells were lysed. (**C**) U2392 cells transfected with control (*CTL*) or DDX3X shRNA were lysed. (**D**) HuT78 cells were nucleofected with plasmid containing wild-type DDX3X (*DDX3X OE*) or vector alone and then lysed after 72 h. (**E**) U2392 cells were nucleofected with plasmid containing DDX3X OE or vector alone and then lysed after 48 h. All the cellular lysates (as indicated in the figure panels) were Western immunoblotted for quantifying the expression levels of vimentin. Blots were re-probed for GAPDH as loading control. Data represent at least 3 independent experiments. Values are mean ± SEM; **, *p* < 0.01 *, *p* < 0.05. **Supplementary Fig. 5.** Gene-set enrichment analysis (GSEA) of DDX3X-mutant/depleted DLBCL, CTCL and NKTCL cells. Genomic data from DDX3X-R475C mutant U2392 (**A**), DDX3X-depleted HuT78 (**B**), and DDX3X-depleted SNK1 (**C**) cells were analyzed by GSEA. Hallmark gene sets (H) and oncogenic signature (C6) gene sets were collected from the Molecular Signatures Database (MSigDB) to produce the enrichment plots. *p*-values were calculated by 1000-gene-set two-sided permutation tests. **Supplementary Fig. 6.** Upregulation of cyclin-D1 in BL cells upon DDX3X depletion. Control and DDX3X-depleted Raji cells (using *shRNA#1* or *shRNA#2*) were lysed, and cellular lysates were analyzed for the expression levels of DDX3X and cyclin-D1 by Western immunoblotting. Blots were re-probed with anti-β-actin to confirm equal loading. Data represent at least 3 independent experiments. Values in the relative densitometry graphs are mean ± SEM; *, *p* < 0.05. **Supplementary Fig. 7.** Effect of DDX3X loss on STAT3 regulation in CTCL cells. (**A**) HuT78 cells were treated with DDX3X siRNA and STAT3 GapmeR. Cellular lysates were collected and Western immunoblotted to determine the expression levels pSTAT3(Y705), STAT3, DDX3X, and GAPDH (loading control). Relative densitometry graph of immunoblots is presented. *, *p* < 0.01. (**B**) Cell lysates of HuT78 cells were immuno-precipitated (*IP*) using anti-DDX3X antibody or IgG control. Subsequently the immuno-precipitates were resolved on SDS-PAGE and subjected to immunoblotting with anti-STAT3 and anti-DDX3X antibodies. Data represent at least 3 independent experiments.

**Additional file 2: Supplementary Table 1.** Details of somatic mutations identified in 9/167 patients with DBCL.

**Additional file 3: Supplementary Table 2.** A list of mutated genes that co-existed in DLBCL patients with DDX3X mutations. **Supplementary Table 3.** A list of DEGs in DDX3X-R475C mutant vs wild-type U2932 cells. **Supplementary Table 4.** A list of DEGs in DDX3X-depleted vs control HuT78 cells. **Supplementary Table 5.** A list of DEGs in DDX3X-depleted vs control SNK1 cells. **Supplementary Table 6.** Top 10 biological pathways based on DAVID and IPA.

## Data Availability

RNA-seq data is publically available from the NCBI with GEO Accession #GSE163817. Other datasets are provided in the additional files.

## Abbreviations

BL: Burkitt lymphoma
CTCL: Cutaneous T-cell lymphoma
DDX3X: DEAD box helicase 3, X-linked
DLBCL: Diffuse large B-cell lymphoma
HDAC: Histone deacetylase
NHL: Non-Hodgkin lymphoma
NKTCL: Natural Killer/T-cell lymphoma
