# Supplementary material for: DDX3X loss is an adverse prognostic marker in diffuse large B-cell lymphoma and is associated with chemoresistance in aggressive non-Hodgkin lymphoma subtypes
Source: Mol Cancer. 2021 Oct 16;20:134. doi: 10.1186/s12943-021-01437-0 (PMC8520256; doi:10.1186/s12943-021-01437-0)
Supplement: Supplementary file 1 — Additional file 1: Supplementary Fig. 1. RNAi-mediated depletion of DDX3X in selected NHL cell lines. The above indicated NHL cell lines and primary T-cells were nucleofected with DDX3X siRNA or Mock siRNA (control) and incubated for 72 h. Few cell lines were stably transfected with DDX3X shRNA or control (CTL) shRNA (as indicated) and were induced to knockdown DDX3X. In addition, U2392 cells were induced to express mutant DDX3X-R475C using CRISPR knock-in technique. (A) Cellular lysates from control and DDX3X-depleted/mutant cells were Western immunoblotted for quantifying DDX3X protein expression. Blots were re-probed for GAPDH or β-actin as loading control. (B) Relative levels of DDX3X mRNA were evaluated using quantitative reverse transcription PCR (RT-qPCR). Values in the RT-qPCR bar graphs are mean ± SEM. Results represent at least 3 independent experiments. **, p < 0.01. Supplementary Fig. 2. Consequence of DDX3X loss on refractoriness of HuT78 cells towards the effect of vorinostat. HuT78 cells were nucleofected with DDX3X siRNA or Mock siRNA (control) and after 48 h, cells were incubated with vorinostat (IC50). Cells were analyzed using MTS assay for cell viability (A) and Annexin-V/PI staining with subsequent flow-cytometry for apoptosis (B). Cell lysates were collected and Western immunoblotted for determining the expression levels of DDX3X, cleaved PARP, and cleaved caspase 3 (C). Blots were re-probed with anti-GAPDH to confirm equal loading. Values are mean ± SEM and results represent at least 3 independent experiments. **, p < 0.01; ***, p < 0.001; ****, p < 0.0001. Supplementary Fig. 3. Effect of DDX3X overexpression on the rate of proliferation of CTCL and DLBCL cells. HuT78 and U2392 cells were nucleofected with plasmid containing wild-type DDX3X (DDX3X OE) or vector alone. The overexpression of DDX3X in HuT78 (A) and U2392 (B) cells was confirmed by Western immunoblotting. Relative densitometry graphs of DDX3X in both cell types are shown. (C) The [file 12943_2021_1437_MOESM1_ESM.pdf]

Supplementary Figures

DDX3X Loss is an Adverse Prognostic Marker in Diffuse Large B-Cell Lymphoma and is Associated with Chemoresistance in Aggressive Non-Hodgkin Lymphoma Subtypes

Atish Kizhakeyil, Nurmahirah Binte Mohammed Zaini, Zhi Sheng Poh, Brandon Han Siang Wong, Xinpeng Loh, Aik Seng Ng, Zun Siong Low, Praseetha Prasannan, Chun Gong, Michelle Guet Khim Tan, Chandramouli Nagarajan, Dachuan Huang, Pang Wan Lu, Jing Quan Lim, Sharon Barrans, Choon-Kiat Ong, Soon-Thye Lim, Wee-Joo Chng, George Follows, Daniel J Hodson, Ming-Qing Du, Yeow Tee Goh, Suat Hoon Tan, Nicholas Francis Grigoropoulos, Navin Kumar Verma

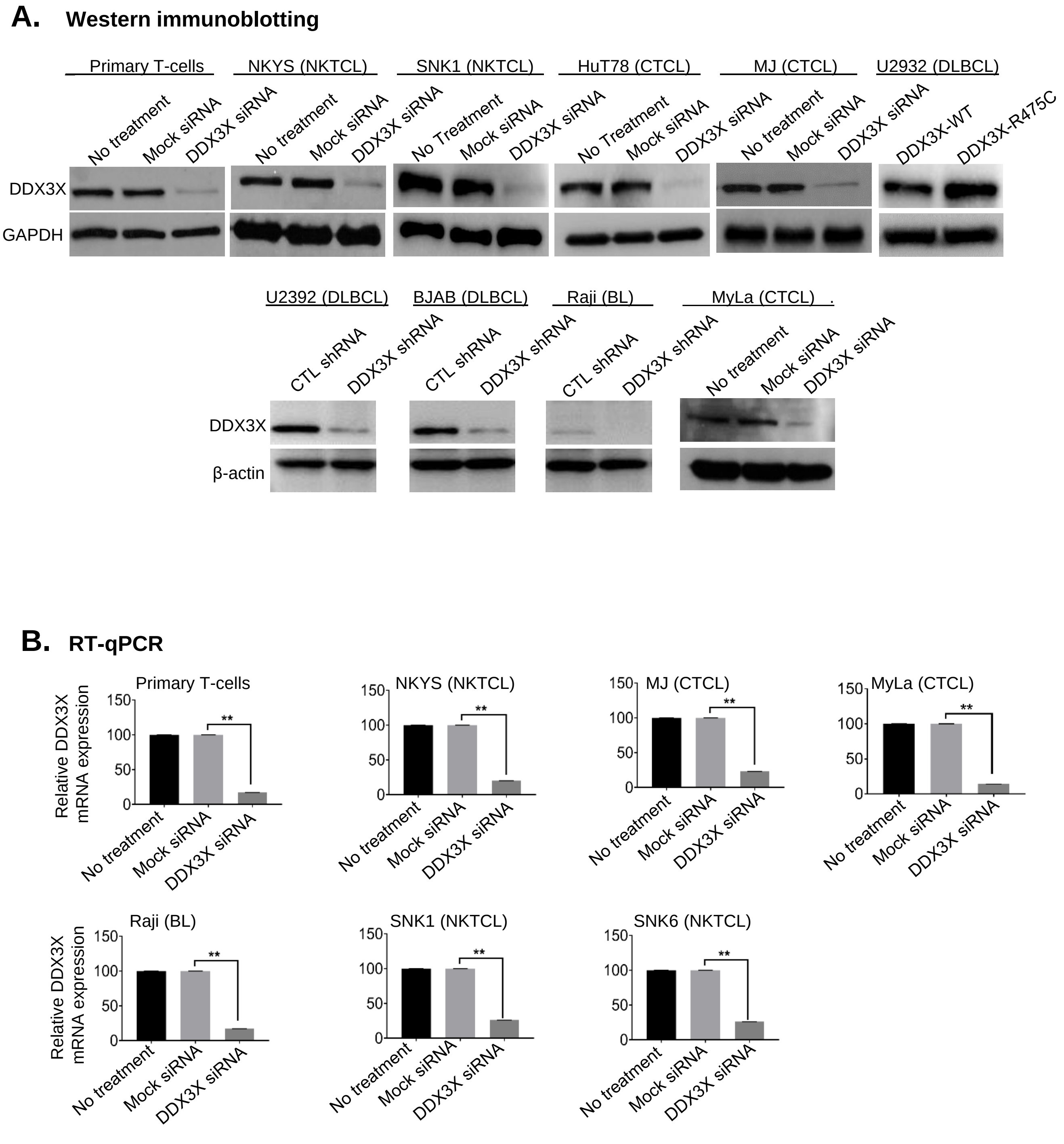

Figure S1. RNAi-mediated depletion of DDX3X in selected NHL cell lines. The above indicated NHL cell lines and primary T-cells were nucleofected with DDX3X siRNA or Mock siRNA (control) and incubated for 72 h. Few cell lines were stably transfected with DDX3X shRNA or control (*CTL*) shRNA (as indicated) and were induced to knockdown DDX3X. In addition, U2392 cells were induced to express mutant DDX3X-R475C using CRISPR knock-in technique. (**A**) Cellular lysates from control and DDX3X-depleted/mutant cells were Western immunoblotted for quantifying DDX3X protein expression. Blots were re-probed for GAPDH or β-actin as loading control. (**B**) Relative levels of DDX3X mRNA were evaluated using quantitative reverse transcription PCR (RT-qPCR). Values in the RT-qPCR bar graphs are mean ± SEM. Results represent at least 3 independent experiments. **, $p < 0.01$.

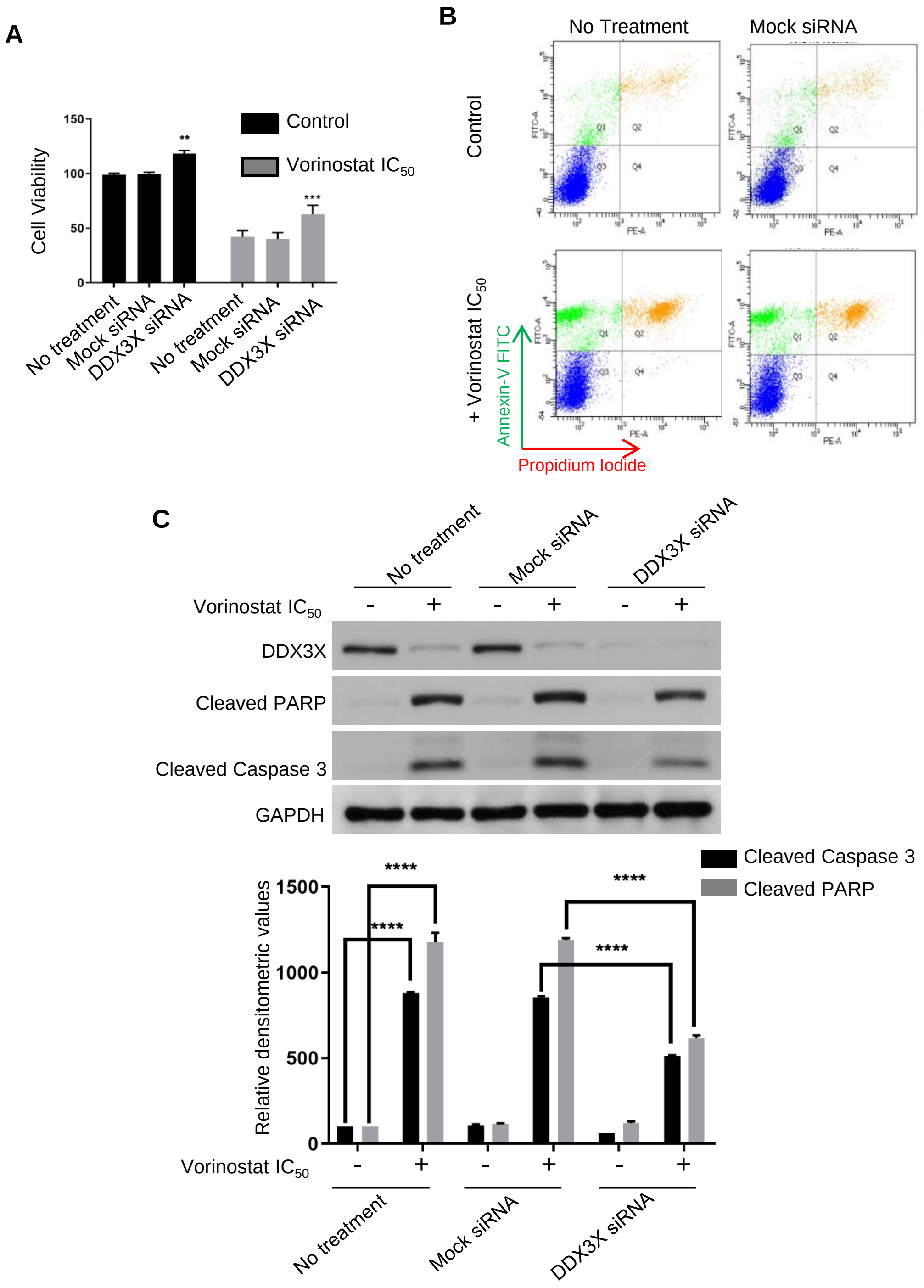

Figure S2. Consequence of DDX3X loss on refractoriness of HuT78 cells towards the effect of vorinostat. HuT78 cells were nucleofected with DDX3X siRNA or Mock siRNA (control) and after 48 h, cells were incubated with vorinostat (IC_{50}). Cells were analyzed using MTS assay for cell viability (**A**) and Annexin-V/PI staining with subsequent flow-cytometry for apoptosis (**B**). Cell lysates were collected and Western immunoblotted for determining the expression levels of DDX3X, cleaved PARP, and cleaved caspase 3 (**C**). Blots were re-probed with anti-GAPDH to confirm equal loading. Values are mean ± SEM and results represent at least 3 independent experiments. **, p<0.01; ***, p<0.001; ****, p<0.0001.

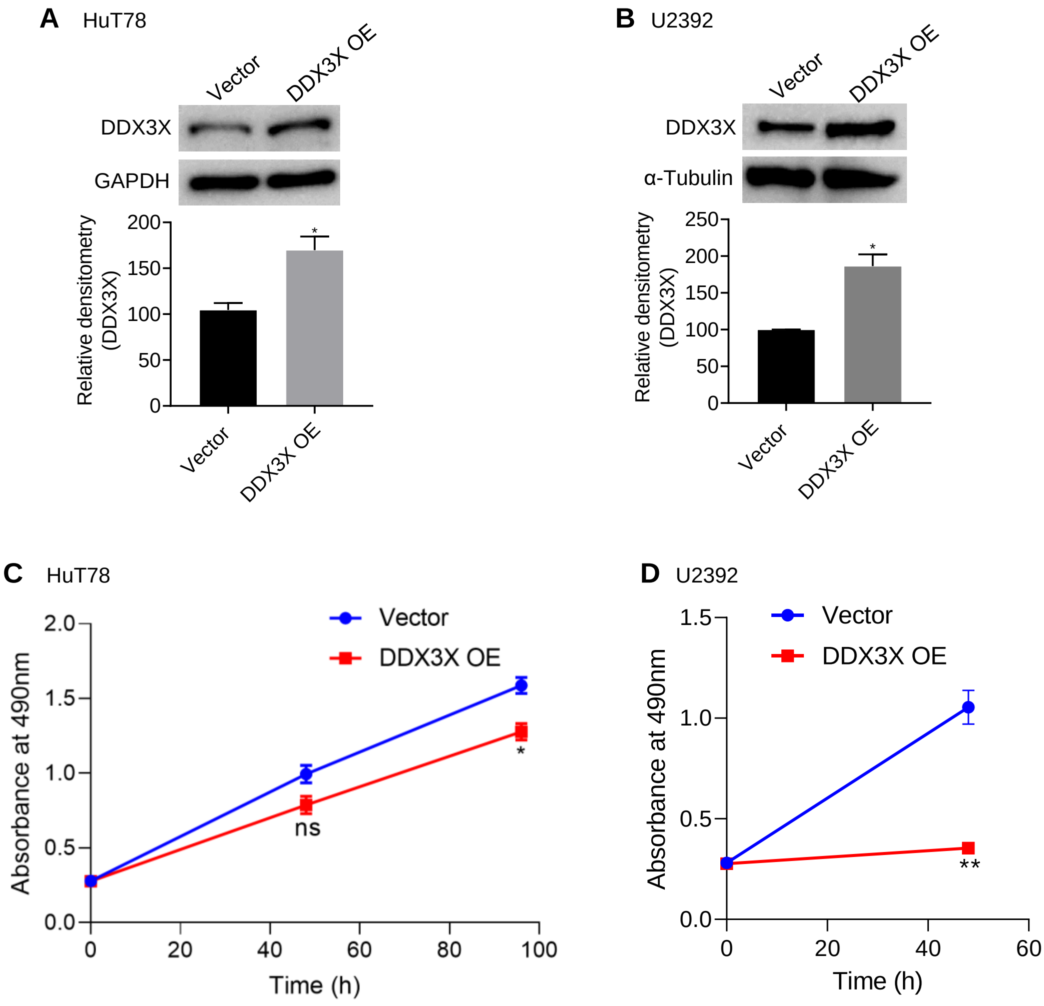

Figure S3. Effect of DDX3X overexpression on the rate of proliferation of CTCL and DLBCL cells. HuT78 and U2392 cells were nucleofected with plasmid containing wild-type *DDX3X* (*DDX3X OE*) or vector alone. The overexpression of DDX3X in HuT78 (**A**) and U2392 (**B**) cells was confirmed by Western immunoblotting. Relative densitometry graphs of DDX3X in both cell types are shown. (**C**) The rate of HuT78 cell proliferation was determined by MTS-based assay, performed in triplicates at 48 h and 96 h. (**D**) The viability of U2392 cells was determined by MTS-based assay, performed in triplicates at 48 h. Values are mean ± SEM and results represent at least 3 independent experiments. *, $p<0.05$; **, $p<0.01$.

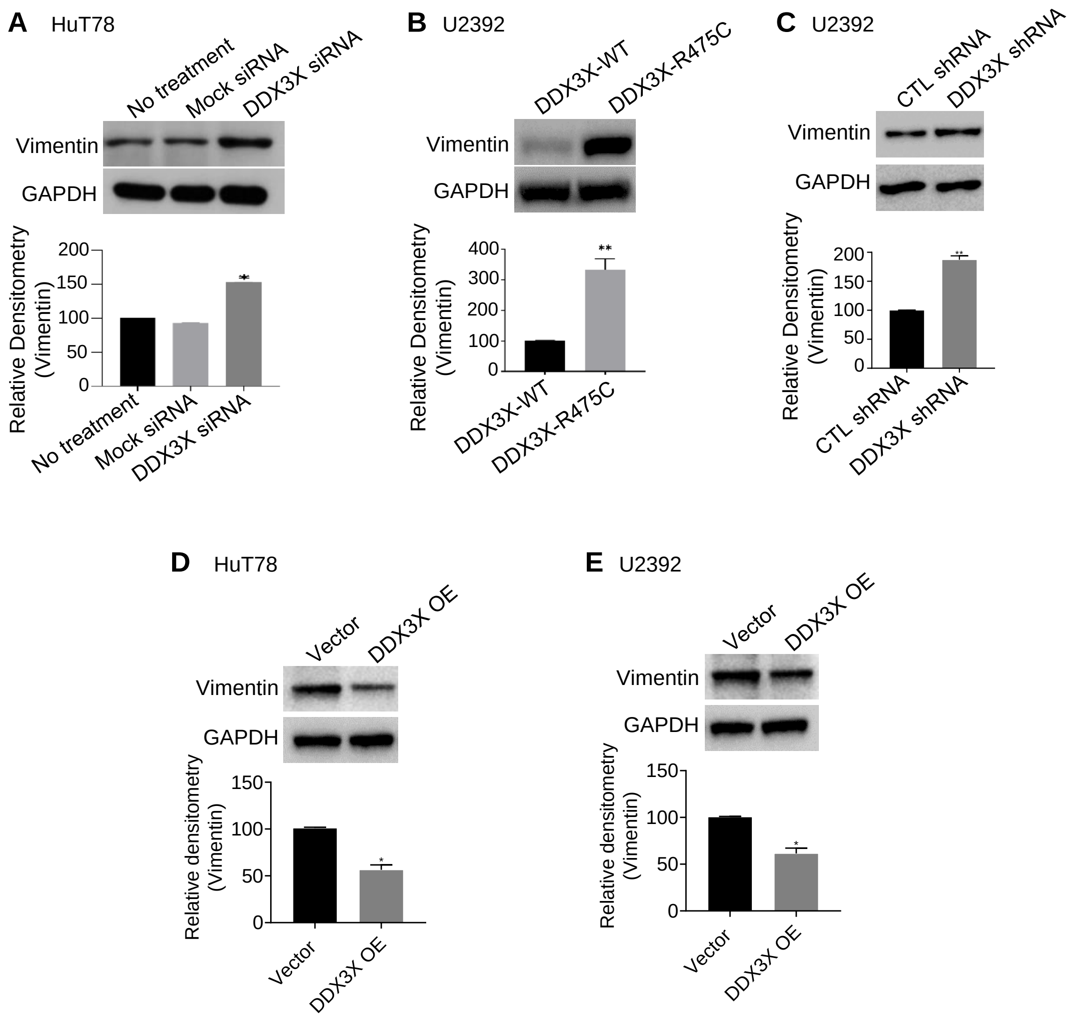

Figure S4. Effect of the modulation of DDX3X expression on the expression levels of vimentin in CTCL and DLBCL cells. (**A**) HuT78 cells were nucleofected with DDX3X siRNA or Mock siRNA (control) and then lysed after 72 h. (**B**) DDX3X wild-type (*WT,* control) and CRISPR knock-in mutant DDX3X-R475C U2392 cells were lysed. (**C**) U2392 cells transfected with control (*CTL*) or DDX3X shRNA were lysed. (**D**) HuT78 cells were nucleofected with plasmid containing wild-type *DDX3X* (*DDX3X OE*) or vector alone and then lysed after 72 h. (**E**) U2392 cells were nucleofected with plasmid containing *DDX3X OE* or vector alone and then lysed after 48 h. All the cellular lysates (as indicated in the figure panels) were Western immunoblotted for quantifying the expression levels of vimentin. Blots were re-probed for GAPDH as loading control. Data represent at least 3 independent experiments. Values are mean ± SEM; **, p<0.01 *, p<0.05.

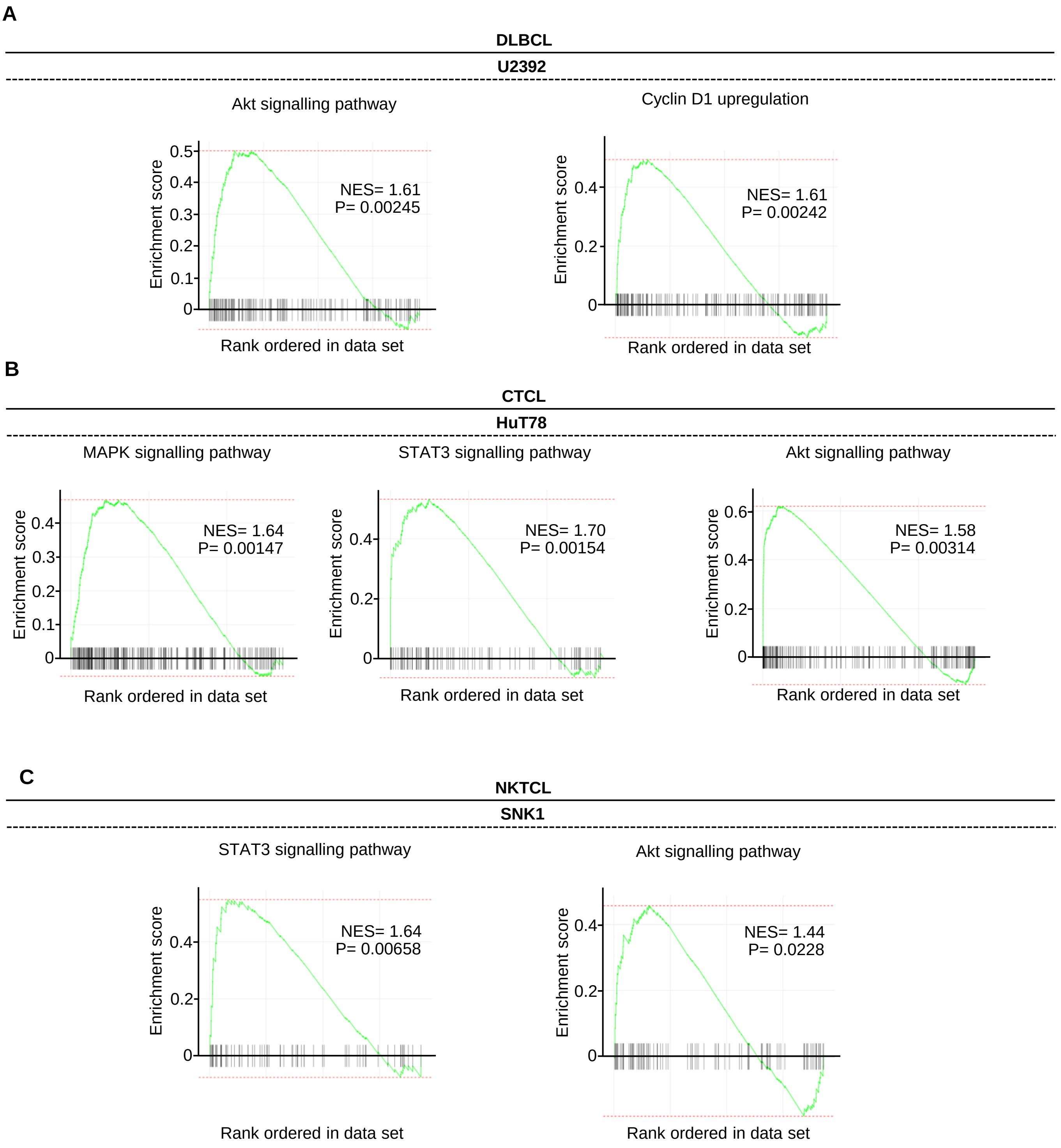

Figure S5. Gene-set enrichment analysis (GSEA) of DDX3X-mutant/depleted DLBCL, CTCL and NKTCL cells. Genomic data from DDX3X-R475C mutant U2392 **(A)**, DDX3X-depleted HuT78 **(B)**, and DDX3X-depleted SNK1 **(C)** cells were analyzed by GSEA. Hallmark gene sets (H) and oncogenic signature (C6) gene sets were collected from the Molecular Signatures Database (MSigDB) to produce the enrichment plots. p-values were calculated by 1,000-gene-set two-sided permutation tests.

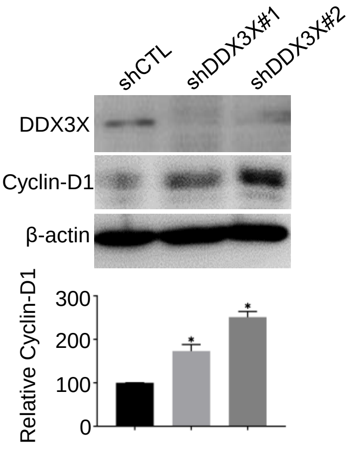

Figure S6. Upregulation of cyclin-D1 in BL cells upon DDX3X depletion. Control and DDX3X-depleted Raji cells (using shRNA#1 or shRNA#2) were lysed, and cellular lysates were analyzed for the expression levels of DDX3X and cyclin-D1 by Western immunoblotting. Blots were re-probed with anti-β-actin to confirm equal loading. Data represent at least 3 independent experiments. Values in the relative densitometry graphs are mean ± SEM; *, $p<0.05$.

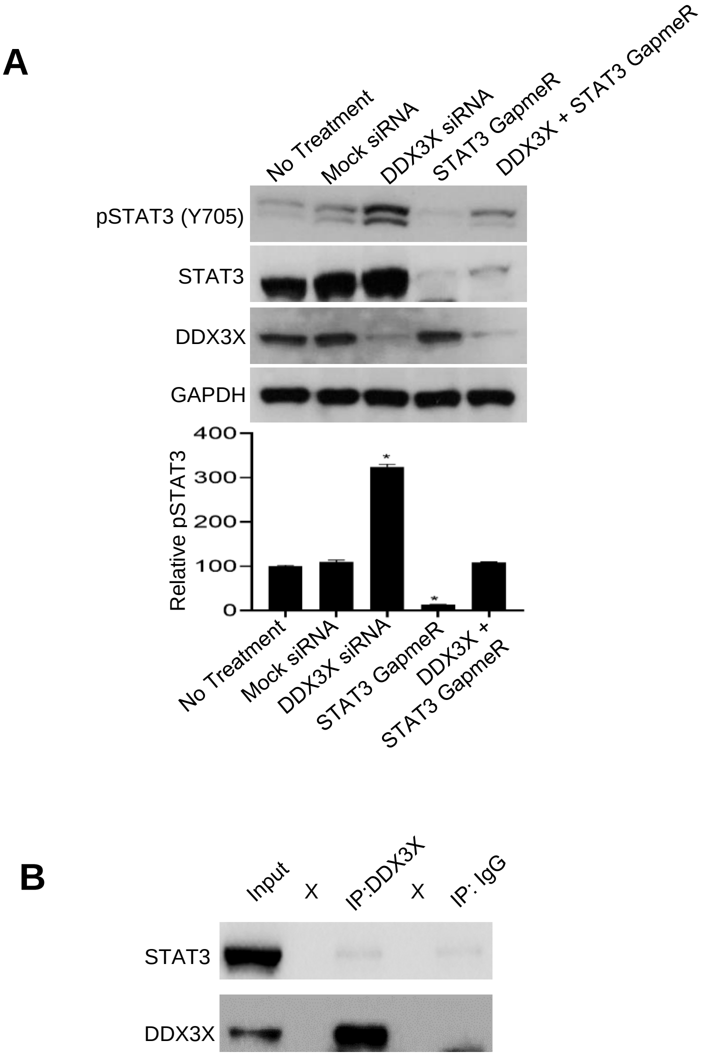

Figure S7. Effect of DDX3X loss on STAT3 regulation in CTCL cells. (**A**) HuT78 cells were treated with DDX3X siRNA and STAT3 GapmeR. Cellular lysates were collected and Western immunoblotted to determine the expression levels pSTAT3(Y705), STAT3, DDX3X, and GAPDH (loading control). Relative densitometry graph of immunoblots is presented. *, p<0.01. (**B**) Cell lysates of HuT78 cells were immuno-precipitated (*IP*) using anti-DDX3X antibody or IgG control. Subsequently the immuno-precipitates were resolved on SDS-PAGE and subjected to immunoblotting with anti-STAT3 and anti-DDX3X antibodies. Data represent at least 3 independent experiments.
