# Supplementary material for: DDX3X loss is an adverse prognostic marker in diffuse large B-cell lymphoma and is associated with chemoresistance in aggressive non-Hodgkin lymphoma subtypes
Source: Mol Cancer. 2021 Oct 16;20:134. doi: 10.1186/s12943-021-01437-0 (PMC8520256; doi:10.1186/s12943-021-01437-0)
Supplement: Supplementary file 2 — Additional file 2: Supplementary Table 1. Details of somatic mutations identified in 9/167 patients with DBCL. [file 12943_2021_1437_MOESM2_ESM.docx]

**Supplementary Table S1.** Details of somatic mutations identified in 9/167 patients with DBCL.

| **Case ID** | **Age/Sex** | **MYC/BCL2/BCL6 translocation status** | **TP53 mutation status** | **DDX3X mutations in DLBCL** | | | | |
| --- | --- | --- | --- | --- | --- | --- | --- | --- |
|  |  |  |  | **Nucleotide change** | **AAF** | **Amino acid change** | **Domain affected** | **PolyPhen score** |
| WES1 | 53/F | MYC trans +ve, BCL2 trans +ve, BCL6 trans -ve | WT | c.1423C>T | 0.49 | R475C | Helicase C -terminal domain | 1 |
|  |  |  |  | c.598T>C | 0.14 | Y200H | -- | 1 |
| WES3 | 59/M | MYC trans -ve, BCL2 trans +ve, BCL6 trans -ve | WT | c.1424G>A | 0.69 | R475H | Helicase C -terminal domain | 1 |
| WES4 | 52/M | MYC trans +ve, BCL2 trans +ve, BCL6 trans -ve | R273C | c.1118_1119insG | 0.3 | V375fs*8 | Helicase ATP binding domain | Frameshift |
| WES7 | 55/F | MYC trans -ve, BCL2 trans -ve, BCL6 trans -ve | WT | c.886C>T | 0.26 | R296C | Helicase ATP binding domain | 1 |
|  |  |  |  | c.1601G>A | 0.31 | R534H | Helicase C -terminal domain | 1 |
| L0022 | -- | BCL6 translocation  BCL6 amplification | WT | c.1924T>G | 0.45 | F357V | Helicase C -terminal domain | 0.996 |
| L0062 | -- | BCL6 translocation | WT | c.1924T>G | 0.40 | F357V | Helicase C -terminal domain | 0.996 |
| L0080 | -- | BCL2 gain | WT | c.2264G>A | 0.11 | S470N | Helicase ATP binding domain | 0.999 |
| L0110 | -- | Normal | S241T | c.1781A>G | 0.61 | Q309R | Helicase ATP binding domain | 1 |
| L0145 | -- | Myc and BCL2 double hit | WT | c.2558C>T | 0.70 | P568L | -- | 1 |

Note: Data is from 4/9 patients with R/R-DLBCL subjected to whole genome sequencing (WES) and 5/158 unselected patients with DLBCL sequenced with a targeted gene panel**.**
